# Screening of aptamers specific to colorectal cancer cells and stem cells by utilizing On-chip Cell-SELEX

**DOI:** 10.1038/srep10326

**Published:** 2015-05-22

**Authors:** Lien-Yu Hung, Chih-Hung Wang, Yu-Jui Che, Chien-Yu Fu, Hwan-You Chang, Kuan Wang, Gwo-Bin Lee

**Affiliations:** 1 Department of Power Mechanical Engineering, National Tsing Hua University, Hsinchu, Taiwan 30013; 2 Institute of Molecular Medicine, National Tsing Hua University, Hsinchu, Taiwan 30013; 3 Institute of Biological Chemistry, Academia Sinica, Taipei, Taiwan 11529; 4 Institute of NanoEngineering and Microsystems, National Tsing Hua University, Hsinchu, Taiwan 30013; 5 Institute of Biomedical Engineering, National Tsing Hua University, Hsinchu, Taiwan 30013

## Abstract

Colorectal cancer (CRC) is the most frequently diagnosed cancer around the world, causing about 700,000 deaths every year. It is clear now that a small fraction of CRC, named colorectal cancer stem cells (CSCs) exhibiting self-renewal and extensive proliferative activities, are hard to be eradicated. Unfortunately, highly specific biomarkers for colorectal CSC (CR-CSCs) are lacking that prohibits the development of effective therapeutic strategies. This study designed and manufactured a novel microfluidic system capable of performing a fully automated cell-based, systematic evolution of ligands by exponential enrichment (SELEX) process. Eight CR-CSC/CRC-specific aptamers were successfully selected using the microfluidic chip. Three of the aptamers showed high affinities towards their respective target cells with a dissociation constant of 27.4, 28.5 and 12.3 nM, which are comparable to that of antibodies.

Cancer is one of the leading causes of death in humans. Colorectal cancer (CRC) is the most frequently diagnosed cancer claiming about 700,000 lives every year^1^. The earlier the cancer is diagnosed, a significantly increase in the five-year survival rate of the patients is observed. For example, patients diagnosed with stage I CRC have a five-year survival rate higher than 90%. The number drops to less than 10% at stage IV reflecting the importance of early diagnose of CRC^2^.

Traditional methods for CRC diagnosis commonly involved invasive approaches such as digital rectal examination, proctoscopy, flexible sigmoidoscopy, and colofibroscopy. These endoscopy-based methods are generally accurate tests offering advantages such as direct observation of polyps and therefore are wildly used in hospitals. Like other invasive diagnosis methods, these approaches possess a higher risk and can result in discomfort^3^. Fecal occult blood test (FOBT) is a cheap and simple to perform method, although the false-positive result is generally high^3^,^4^,^5^. Furthermore, serological tests using carcinoembryonic antigen (CEA) and carbohydrate antigen 19-9 (CA19-9) as biomarkers for CRC diagnosis have also been performed^6^,^7^. However, these markers are not specific enough for CRC early detection since patients with pancreatic cancer and lung cancer also show an increase of CEA and CA 19-9 values.

The advancement of cancer therapeutic technology has greatly improved the survival rates of patients with CRC, although recurrence of the cancer is still common^8^. It is recognized now that a small fraction of cancer cells, named cancer stem cells (CSC), show distinct biological features from other cells in the cancer population^9^,^10^,^11^. Cancer stem cells possess the ability of self-renewal, the capability of developing multiple cell lineages, and the potential of extensive proliferation. Cancer stem cells also display high drug resistance and are therefore difficult to eradicate^11^,^12^,^13^. If therapies can be targeted against CSCs such that the tumor may lose its ability of growing and maintaining, then it may eventually lead to a complete cure^14^. Cancer stem cells have been identified in CRC^15^, and the cells are known to contribute to metastasis in the patients after receiving chemotherapy^16^. In order to detect or isolate CR-CSCs, certain cell surface molecules including CD44, CD133 (Prominin-1), and EpCAM have been used as biomarkers of CR-CSCs^11^,^17^,^18^,^19^,^20^,^21^. However, these molecules are also present in other types of CSCs and do not have sufficient specificity for CR-CSC detection^12^,^22^,^23^,^24^. Therefore, the development of a technology to efficiently identify novel specific biomarkers for CR-CSC and CRC cells detection will contribute greatly in diagnosis and treatment of CRC.

In this study, we propose a new approach for screening aptamer targeting agents for CR-CSC and CRC. An *in-vitro* marker screening method, systematic evolution of ligands by exponential enrichment (SELEX), has already been used for screening different targets ranging from small chemical molecules, proteins too even whole cells^25^,^26^,^27^,^28^. With this *in-vitro* marker screening method, we may screen different tumor markers specific for the CR-CSC and CRC^28^,^29^. However, the SELEX-based screening method requires a long time and sophisticate skills to complete, and consumes relatively large quantity of specimens and reagents. Recently, SELEX processes operated on a microfluidic chip, providing advantages such as rapid, high-throughput and high-efficiency, have been tested. For example, CE-SELEX systems^30^,^31^, sol-gel isolation SELEX systems^32^ and magnetic-bead-based SELEX systems^33^ have been demonstrated. An automatic microfluidic system for screening of aptamers specific to the CSC associated with lung cancer was also developed by our group^34^.

This study therefore presents a new integrated microfluidic system for continuous selection of aptamers specific to the CR-CSC and CRC using a cell-based SELEX (Cell-SELEX) process. When compared with our previous study^35^, first, this new system used colorectal cancer stem cells for aptamer targeting agents screening, which has never been explored. Second, the chip design was greatly simplified since the screening process was performed in a circular layout while it was a linear layout in our previous work. With this approach, the on-chip PCR chamber was connected with a transportation unit for continuously performing the screening process such that the whole system was relatively compact in size. Third, less manual operation was involved in the screening process, which makes this developed system become a “micro-total-analysis-system” for automatic Cell-SELEX. In this study, eight CR-CSC/CRC-specific aptamers were successfully selected. Three of them showed high affinities towards their target cells with dissociation constants (K_d_) of 27.4, 28.5 and 12.3 nM. Furthermore, specific aptamer-stained fluorescence microscopic images and a competitive testing against other cancer cell lines were examined to verify the high affinity and selectivity of these selected CR-CSC/CRC aptamers. These results demonstrated that the integrated microfluidic system is able to rapidly screen aptamers with high affinity and selectivity within a shorter period of time. This technology is promising for screening of aptamer targeting agents for these cancer cells, which could be useful for early diagnosis of CR-CSC/CRC or even target therapeutics.

## Results

### Characterization of the tumorspheres cultured CR-CSCs with specific biomarkers

**Figure 1:**
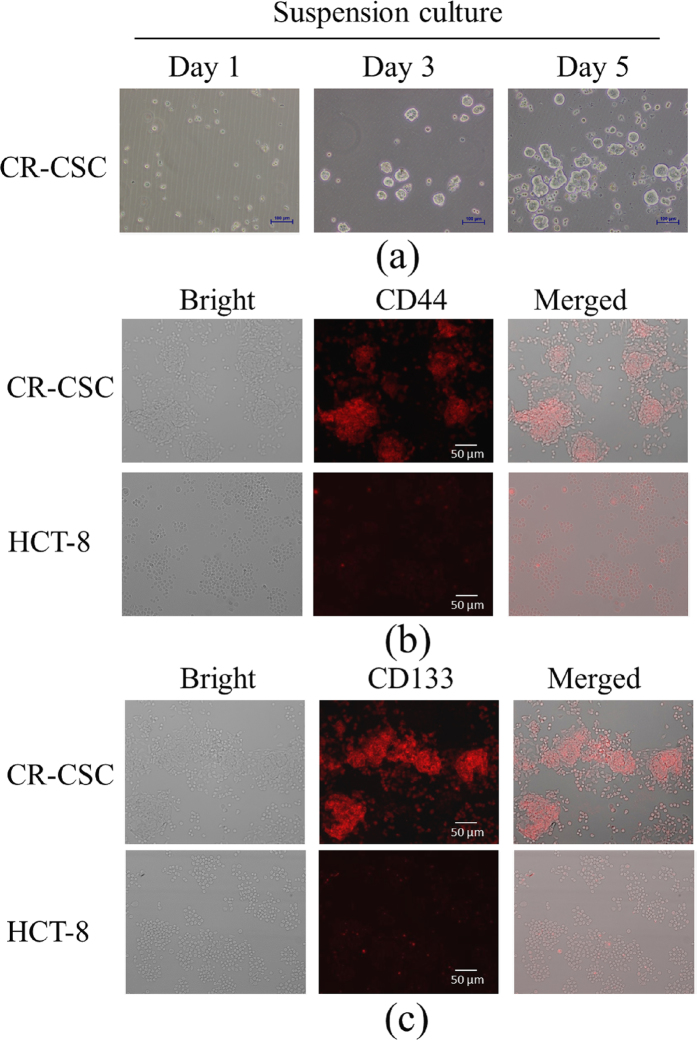
(**a**) CR-CSCs form suspension cultured tumorspheres from culture day one to day five; (**b**) anti-CD44 fluorescence immunostaining analysis with CR-CSCs after enrichment suspension culture and colorectal cancer cells (HCT-8); (**c**) anti-CD133 fluorescence immunostaining analysis with CR-CSCs after enrichment suspension culture and HCT-8.

Tumorspheres cultured CR-CSCs and HCT-8 cells were used as target cells and control cells, respectively. Figure 1(a) shows that CR-CSCs form suspension cultured tumorspheres from culture day one, and grow up to day five. Figure 1(b) shows that anti-CD44 fluorescence immunostaining analysis with CR-CSCs after enrichment suspension culture and HCT-8; In Fig. 1(c), anti-CD133 fluorescence immunostaining analysis with CR-CSCs after enrichment suspension culture and HCT-8 anti-CD44 specific antibody and anti-CD133 specific antibody were first used to confirm CR-CSCs. These fluorescence images of HCT-8 and CR-CSC indicated that only CR-CSCs presented red fluorescence signals significantly, indicating that these cells expressed abundant CD44 and CD133 proteins and had CR-CSC properties.

### Cell-SELEX working process and chip design

**Figure 2:**
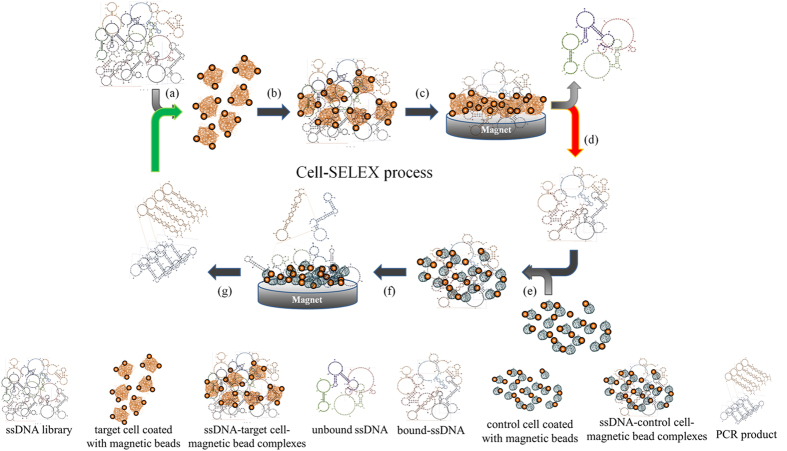
The Cell-SELEX process for screening of aptamers specific to CR-CSCs and CRC includes: (**a**) adding ssDNA and target cells in the incubation chamber, (**b**) mixing ssDNA with target cells, (**c**) using a magnet to collect the bound ssDNA-target cell-magnetic bead complexes and washing away unbound ssDNA, (**d**) heating to lyse the target cells and releasing the bound ssDNA, (**e**) mixing the released ssDNA with control cells, (**f**) applying a magnet to collect the bound ssDNA-control cell-magnetic bead complexes and collecting the unbound ssDNA as aptamer candidates, and (**g**) performing PCR to amplify the selected ssDNA for next round of CR-CSC Cell-SELEX screening.

**Figure 3:**
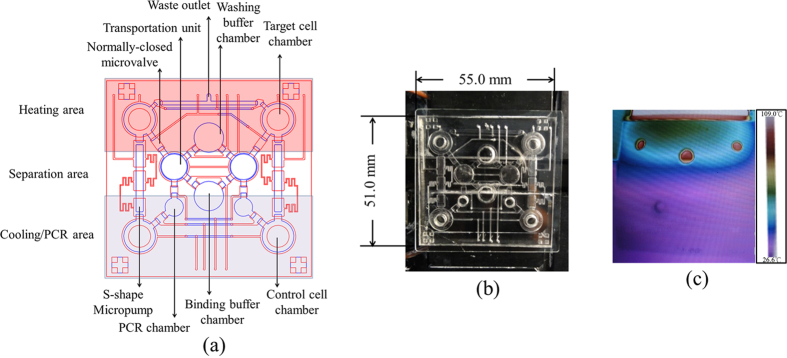
(**a**) The layout of the integrated microfluidic chip equipped with multiple micro-devices, including washing buffer and the binding buffer loading chambers, two transportation units, normally-closed microvalves, open-chamber micromixers/micropumps, serpentine-shape (S-shape) micropump and PCR chambers. The red color defines the heating area and the purple color represents the cooling/PCR area, and a separation area is located between them. (**b**) A photograph of the chip with a size of 51.0 mm × 55.0 mm. (**c**) An infrared image of the heating and cooling blocks with a temperature distribution ranging from 26.6 °C (purple) to 109.0 °C (red).

The working process of CR-CSC Cell-SELEX is schematically shown in Fig. 2 using CRC line HCT-8 and its derivative CR-CSC as a model for specific aptamer screening. The detailed working procedure of the on-chip aptamer selection is shown in Supplemental Information Table S1. Furthermore, Fig. 3(a) shows a schematic top-view of the chip, which was equipped with one sample transportation unit, two reagent loading chambers, normally-closed microvalves, two target cell chambers, two control cell chambers, two serpentine-shape micropumps (S-shape micropump), and two PCR chambers for the final amplification step of the aptamer candidate and further thermal denature. The relationship between the pumping rate and the driving frequency of the transportation unit and the S-shape micropump could be found in Supplemental Information Figures S1(a) and S1(b). Figure 3(b) is a photograph of the integrated microfluidic chip placed on a custom-made dual-temperature control module composing of two Peltier cooling/heating units. Furthermore, the dimensions of the chip were measured to be 51.0 mm × 55.0 mm. Figure 3(c) shows the temperature distributions measured by an infrared imaging camera and the details of this chip are shown in Methods section. It is important to note that the PCR chamber was designed to connect with a transportation unit. Thus, the PCR products of each screening round would be automatically transported to the target cell chamber for the next round of SELEX screening. This kind of chip design improved the automation of the on-chip SELEX process by connecting the last step (PCR amplification) to the very first step (adding denatured-PCR products to target cells).

### Binding assays of the enriched ssDNA pools with CR-CSCs and HCT-8 cells

**Figure 4:**
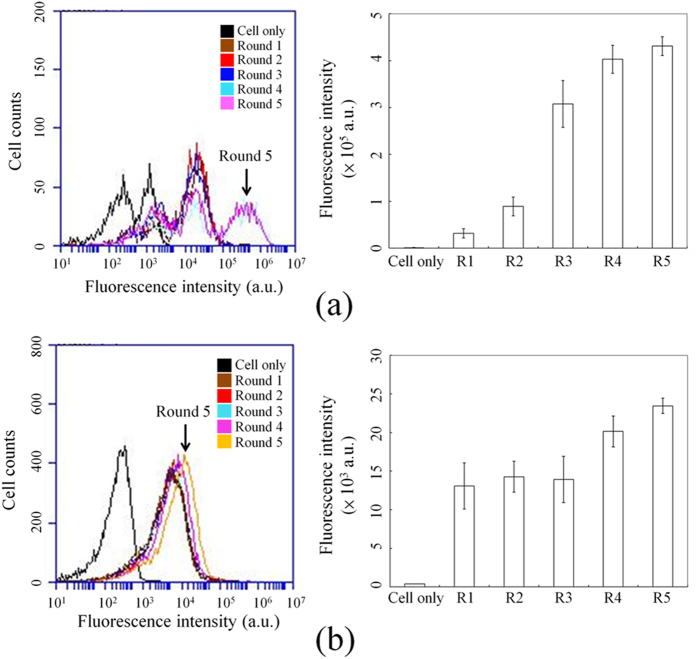
Flow cytometric analysis of the screened ssDNA pools after five rounds (R1 to R5) of on-chip CSC Cell-SELEX. (**a**) Screened products from the selection rounds while using CR-CSC as a target cell; (**b**) Screened products from the selection rounds when using HCT-8 as a target cell. The line colors are defined as black, cell only; brown, the first round (R1); red, the second round (R2) tests; blue, the third round (R3); light blue, the fourth round (R4); pink, the fifth round (R5). All measurements were conducted three times.

The selected ssDNA pools for each round of Cell-SELEX, from first round (R1) to the fifth round (R5), were amplified by PCR. After the PCR, the amplified products were collected, fluorescence labeled, and the enrichment of aptamers with CRC and CR-CSC binding specificity was determined with flow cytometry. When the selected ssDNA pools were specifically enriched and bound primarily to either CR-CSC or HCT-8, the fluorescence intensity would increase from R1 to R5. Figure 4(a) shows that the peak fluorescence signal of the selected ssDNA pools in the fourth round (R4) and R5 for the CR-CSCs dramatically increased to 10^5^ arbitrary units (a.u.) when compared to R1 (at about 10^4^ a.u.). Similarly, the peak fluorescence signal from the R1 to R5 of selected ssDNA pool for the HCT-8 cell line also increased from 10^3^ a.u. to 10^4^ a.u., as shown in Fig. 4(b), indicating the affinity of selected aptamers were increased from round 1 to round 5.

### Identification of selected aptamers specific to CR-CSCs and HCT-8 cells

In this study, a total of 80 aptamers, forty each selected for both CR-CSCs and HCT-8 cells were chosen randomly for further competitive tests to determine their affinity^34^. After these competitive assays, four CR-CSC specific aptamers (CSC-16, CSC-19, CSC-35 and CSC-37) and four HCT-8 specific aptamers (HCT8-15, HCT8-17, HCT8-34 and HCT8-35) were chosen for further testing because they demonstrated a relatively high affinity. These aptamers were then sequenced and their sequences are shown in Table 1. The ssDNA-folding structures of these selected aptamers at 25 °C were further predicted using MFOLD software (version 3.5) and are shown in Supplemental Information Figures S2. The binding affinity of these selected aptamers was further characterized by testing various concentrations of FAM fluorescence modified aptamers and the dissociation constant (K_d_) between each aptamer and their target cells was measured accordingly. Furthermore, CSC-16 showed the highest affinity to CR-CSC with a K_d_ value of 27.4 nM; Similarly, HCT8-17 and HCT8-34 showed the highest affinity to HCT-8 cell line with a K_d_ valve of 28.5 and 12.3 nM.

### Specificity of the selected aptamers

**Figure 5:**
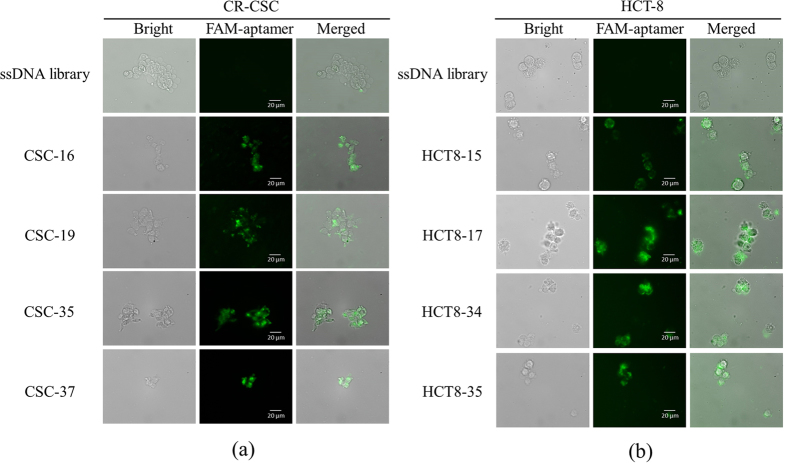
Selected specific aptamer binding analysis by using FAM fluorescence labelled aptamers when binding with their target cells. (**a**) FAM labelled ssDNA library, as a control assay, and CR-CSC specific aptamers, CSC-16, CSC-19, CSC-35, and CSC-37, binding with CR-CSCs; (**b**) FAM labelled ssDNA library, as a control assay, and HCT-8 specific aptamers, HCT8-15, HCT8-17, HCT8-34, and HCT8-35, binding with HCT-8 cells.

In this study, CR-CSC/HCT-8-specific aptamers modified with FAM green fluorescence dye were used to stain CR-CSCs and HCT-8 cell line to determine their specificity. Significant fluorescent signals could be observed on the target cells but not the control cells (Fig. 5). All four CR-CSC specific aptamers could specifically bind to CR-CSCs. Similarly, fluorescence images of HCT-8 cell line stained with aptamers selected specifically for the cells are shown in Fig. 5. A binding control assay was performed in Supplemental Information Figure S3, which showed the incubation of the FAM labelled CR-CSC aptamers with HCT-8 cells, and FAM labelled HCT-8 aptamers with CR-CSC. Most of them showed weak to no green fluorescence signals, indicating that the selected aptamers could only recognize the target cells.

The binding selectivity of CR-CSC/HCT-8-specific aptamers were further tested by examining the interaction between the screened aptamers and other cancer cell lines, including A549, MCF7, BxPC3 HepG2s, HeLa, and NIH3T3 cells. To determine the optimal condition to achieve highly specific interaction between the aptamers and the target cells, several washing steps were conducted after incubating the aptamers and the cells, and the binding of the FAM-aptamers was determined using fluorescence microscopy. As shown in the column “One wash” in Table 2, because only one washing step cannot provide sufficient selection (washing) stringency, the cell capture rates in all these tests by the Dynabeads® Epithelial Enrich immunomagnetic beads (EpiEnrich beads) were relatively higher (>70%). Therefore, in this study, six repeated washings, using the open-chamber micromixers operated at a driving frequency of 1 Hz under -80 kPa for 1-min washing, were applied in order to provide significant stringency for the binding selectivity test.

Four CR-CSC specific aptamers, including CSC-16, CSC-19, CSC-35 and CSC-37, showed relatively high capture rates toward CR-CSCs (target cells) with capture rates of 51.7 ± 5.3%, 52.9 ± 4.7%, 45.6 ± 9.1% and 54.9 ± 4.4%, respectively. Except CSC-16 aptamer which captured MCF-7 and HepG2 (non-target cells) at rates of 32.0 ± 6.5% and 38.1 ± 4.2%, respectively, all other CSC aptamers captured non-target cells at a rate lower than 40%, indicating the high specificity of these aptamers and the usefulness of the Cell-SELEX chip. Similarly, three of four HCT-8 specific aptamers, including HCT8-17, HCT8-34 and HCT8-35, showed relatively high capture rates toward HCT-8 (target cells) with capture rates of 47.8 ± 8.7%, 50.1 ± 1.7%, and 42.5 ± 7.0%, respectively. However, HCT8-15 aptamer could only capture HCT-8 cell with a capture rate of 33.3 ± 2.9%. Both HCT8-17 and HCT8-34 aptamers also captured MCF-7 (non-target cells) with relative high rates (36.1 ± 4.9% and 34.8 ± 3.5%, respectively). Together, the result indicates that the HCT-8 specific aptamers, in particular HCT8-17, HCT8-34 and HCT8-35, can be used to distinguish CRC from CR-CSC. The commercially available, BerEP4 antibody-coated EpiEnriched magnetic beads, significantly high recognition toward CR-CSC, A549 and MCF-7, but a relatively lower recognition to HCT-8 and other cell lines tested in this study were observed.

## Discussion

This study manufactured a new microfluidic system which is capable of performing an automated on-chip Cell-SELEX process. According to these experimental results, the on-chip Cell-SELEX selection could be shortened to only five rounds as the fluorescence signals remained at a stable intensity level between R4 to R5, as shown in Fig. 4. Note that it may take at least 10-20 rounds for traditional Cell-SELEX process to select desired aptamers^28^,^29^. After 5 rounds of Cell-SELEX selections, the PCR products obtained from R5 were cloned by using the TOPO TA cloning kit. Using this microfluidic system, high-affinity aptamers specific to CR-CSCs and HCT-8 cells, respectively could be identified. Note that for CR-CSC selection in Fig. 4, each round exhibited multiple peaks. It is because the monitoring of the enrichment result from the selected ssDNA pools might exhibit the flow peaks in a wide range, which was also reported in previous studies^28^,^36^. Furthermore, the analysis of cancer stem cells needs several biomarkers in flow analysis to show a single peak^37^,^38^. Therefore, it is common to show multiple peaks in this monitoring step of the enrichment result from the selected ssDNA pools, indicating that the analysis of CR-CSC might also need more than one aptamer.

The K_d_ values were measured and found to range from 12.3 nM to 157.2 nM, which is comparable to most antibodies (from 10^−7^ to 10^−9^ M)^39^. Among the eight CR-CSC/CRC-specific aptamers, three of them showed high affinities towards their target cells with dissociation constants of 27.4, 28.5 and 12.3 nM, respectively, comparable to that of antibody-antigen interaction. These results demonstrated that this integrated microfluidic system could be used to select high-affinity aptamers specific to CR-CSCs and HCT-8 cell line. Furthermore, the signal pattern showed that the aptamers were specifically bound onto the surface of each target cell, as shown in Fig. 5. In some cases, the fluorescence signal on the cell membrane could not be clearly defined, indicating that some of the FAM-modified CR-CSC-specific aptamers were transported inside the cell. Furthermore, the FAM modified CSC-19 aptamer was observed to exhibit the weakest signal among four CR-CSC aptamers, reflecting by its relatively high K_d_ value, 157.2 nM. Similarly, the images of the FAM-modified HCT8-17 and HCT8-34 aptamers exhibited more clear fluorescence images than the other two HCT-8-specific aptamers, which is consistent with the fact that HCT8-17 and HCT8-34 aptamers had lower K_d_ values of 28.5 nM and 12.3 nM, respectively. Correspondingly, the fluorescence signals of HCT8-17 and HCT8-34 aptamers could be observed in the region of the cytoplasm, indicating that part of FAM-modified HCT-8-specific aptamers might be taken up into the cell through endocytosis. Note that we only randomly chose 80 aptamers for the competitive tests. There might have some difference between selected aptamer pools and individual aptamer. The aptamers with higher binding affinity might be obtained by integrating next-generation sequencing and bioinformatics analysis for all aptamer candidates.

Furthermore, for CR-CSC cells, EpCAM could be one of its surface markers,^11^,^18^ thus the capture rate of the commercial EpiEnrich beads increased as the selected CR-CSC aptamers. However, the EpiEnrich beads showed lower selectivity than the selected CR-CSC aptamers because the beads captured A549, MCF7, and HeLa cells with relatively high rates (74.0 ± 4.4%, 66.8 ± 6.8%, and 44.4 ± 8.9%, respectively), as shown in Table 2. These results show that this integrated microfluidic system is capable of selecting high-affinity aptamers specific to CR-CSCs and HCT-8 cell line and can also be applied to identifying aptamers specific to other types of cancers. Finally, this developed technique will be useful for screening of aptamer targeting agents for cancer cells or even target therapeutics. In the future, it may be applied in personalized medicine for aptamer screening or drug discovery.

## Methods

### Experimental procedure

The working process is shown in Fig. 2; briefly, a single-stranded DNA (ssDNA) library was incubated with magnetic bead-labelled target cells (Fig. 2(a)) for positive selection (Fig. 2(b)). After magnetic collection and washing out unbound ssDNA (Fig. 2(c)), the ssDNA bound on the target cells were released from the cells by thermal lysis (Fig. 2(d)). The ssDNA was further incubated with control cells to remove non-specific binding aptamers for negative selection (Fig. 2(e)). The incubation supernatant was collected and the ssDNA in the supernatant was amplified using polymerase chain reaction (PCR) (Fig. 2(f)). The amplified ssDNA were used for next round of Cell-SELEX screening (Fig. 2(g)) and the selection cycle was repeated several times until highly specific aptamers were identified. Note that all the steps of incubation, transportation, washing and PCR, with the exception of sample and reagent loading, were performed automatically in the microfluidic system^40^. The target cells and the control cells were applied side-by-side in the system by activating two sets of open-chamber micropumps^40^. Such a design allows selection of aptamers specific to CR-CSCs and CRC cells, respectively to be performed automatically on a single chip.

### Chip design, fabrication process, and micro-devices

In order to rapidly perform the automatic selection of aptamers specific to the CR-CSCs and the HCT-8 cell line by using the Cell-SELEX process, a microfluidic chip was designed and fabricated. It consisted of two polydimethylsiloxane (PDMS, Sylgad 184A/B, Dow Corning Corp., USA) layers and one glass substrate (G-Tech Optoelectronics Corp., Taiwan). A computer-numerical-control (CNC) machining process and a PDMS replication process were applied for microfabrication of a PMMA template which was used as a master mold for the PDMS structures^32^. Finally, by utilizing an oxygen plasma treatment, the PDMS layers and the glass substrate were bonded together to form a sealed microfluidic chip. As shown in Fig. 2(a), the transportation unit^41^, the washing buffer and the binding buffer loading chambers, normally-closed microvalves^42^, four open-chamber micromixers/micropumps^40^, and two PCR chambers. In addition, two S-shape micropump^43^ were designed for transporting the thermally-released ssDNA from the target cell chambers to the control cell chambers, as shown in Fig. 2(a). The infrared imaging camera (HotFind-LG, SDS, UK) identify the temperatures on the dual-temperature control module range from 26.6 °C to 109.0 °C. With the aid of this dual-temperature control module, control cells could be maintained at room temperature such that they would not be damaged before the negative selection process when the target cells were processed in the reaction region at a temperature as high as 95.0 °C during the step to release the bound ssDNA.

In this study, a suction-type transportation unit has been adopted to transport samples and reagents^41^. An open-chamber micromixer/micropump was also designed for sample mixing and transportation in this study. The working principle of the open-chamber micromixer/micropump has been described previously^40^,^41^. Briefly, one set of S-shape micropump was used to transport the ssDNA from the target cell region to the control cell region. The detailed information about this micro-component can be found in our previous work^43^. In brief, these micro-devices were connected to an electromagnetic valve (EMV) that was driven by a custom-made digital controller attached to a vacuum pump and the vacuum force was generated to uplift and release a thin PDMS membrane to transport samples and reagents^36^.

### Single strand DNA library, primers, reagents and PCR operating conditions

The ssDNA library was obtained commercially (Medclub Scientific Co., Ltd., Taiwan) and diluted to the final concentration of 1 μM. Each ssDNA sequence of the library contained a central region of 40 random nucleotide bases flanked by 2 specific 16-base sequences that function as primer-binding sites (a total sequence, i.e. 5’-GGCAGGAAGACAAACA-N_40_-GGTCTGTGGTGCTGT-3’) for the subsequent PCR process. The PCR reagents contained 0.5 μL of deoxynucleotide triphosphates (dNTPs, 0.2 mM each), 2 μL of ssDNA (0.1 μM), 0.125 μL of Taq DNA polymerase (New England Biolabs Co., Ltd., Taiwan), 0.5 μL of forward primers (0.5 μM), and 0.5 μL of reverse primers (0.5 μM). Then double-distilled water (ddH_2_O) was added to make a total volume to 25 μL. The PCR process was performed with an initial denaturation at 95 °C for 10 min, followed by 20 cycles of denaturation at 95 °C for 30 sec, annealing at 63 °C for 15 sec, and extension at 72 °C for 30 sec. A final extension step at 72 °C for 10 min was carried out following the last cycle. The PCR products were heated to 95 °C to denature the double-stranded DNA and were then cooled down rapidly to maintain the denatured DNA in the ssDNA form.

The binding buffer was a mixture of 1 L of Dulbecco’s phosphate-buffered saline (DPBS, Invitrogen Co., USA), 100 mg transfer ribonucleic acid (Sigma Co., USA), 4.5 g of glucose, and 5 mL of 1 M MgCl_2_, and was stored at 4 °C prior to use. A washing buffer containing 1 L of DPBS, 4.5 g of glucose and 5 mL of 1 M MgCl_2_, was stored at 4 °C prior to use^27^. Single strand DNA library, primers, reagents and Each selection round used 300 μL of the washing buffer for the washing process and 50 μL of the binding buffer for incubation of the ssDNA with the cell-magnetic bead complexes. After five rounds of Cell-SELEX screening, the PCR products from the fifth round (R5) were purified and cloned using the TOPO TA Cloning kit (Invitrogen Co., USA). Detail information about TA cloning process could be found in our previous work^34^. Forty colonies of aptamer clones selected for CR-CSCs and for HCT-8 CRC line, respectively were randomly selected and purified, giving a total of 80 colonies. Colonies showing a higher affinity in the competitive tests were nucleotide sequenced and the aptamers were synthesized chemically (Medclub Scientific Co., Taoyuan, Taiwan) and used for the following cell binding experiments to confirm its affinity and specificity to the target cells.

### Tumorsphere culture, adherent cell culture, and preparation of immunomagnetic beads

The CR-CSC was purified from HCT-8 CRC line by a flow cytometric sorting process based on the presence of cell surface marker CD44. The HCT-8 cells were cultured in RPMI-1640 medium (Invitrogen Co., USA). CR-CSCs were cultured in a 6-well ultra-low cluster plate (Costar, Corning Co., France) in 3 mL of CR-CSC medium to form tumorspheres in 7 days. The CSC medium was consisted of serum-free DMEM/F12 medium plus GlutaMAX (Invitrogen Co., USA), 1% N2 supplement (Invitrogen Co., USA), 2% B27 supplement (Invitrogen Co., USA), 50 ng/mL epidermal growth factor (EGF, Sigma-Aldrich Co., USA), 20 ng/mL basic fibroblast growth factor (FGF-b, Sigma-Aldrich Co., USA) and 100 UI/mL penicillin/streptomycin (PS, Invitrogen Co., USA). For dissociation into single cells, tumorspheres were first centrifuged for 3 min at 1000 rpm. The pellet was then gently re-suspended in 1 mL of diluted trypsin-EDTA (Invitrogen Co., USA) before being incubated for 2 min at 37 °C. After adding 2 mL of serum-free DMEM/F12, tumorspheres were dissociated by gentle pipetting and directly transferred back into tumorspheres culture conditions as described above.

A549 (lung cancer cell line), HeLa (cervical cancer cell line), HepG2 (liver cancer cell line) were provided from The Institute of Biomedical Sciences, National Sun Yat-Sen University, Kaohsiung, Taiwan. BxPC3 (pancreas cancer cell line), MCF7 (breast cancer cell line), and NIH3T3 (mouse embryonic fibroblast cell line) were obtained from The Institute of Microbiology and Immunology, Chung Shan Medical University, Taichung, Taiwan. All of these cell lines were cultured in DMEM (Invitrogen Co., USA). All media were supplemented with 10% fetal bovine serum (Invitrogen Co., USA) and PS (100 UI/mL, Invitrogen Co., USA). All cell lines were incubated at 37 °C in a 5% CO_2_ atmosphere.

The Dynabeads® Epithelial Enrich immunomagnetic beads (EpiEnrich, 4 × 10^8^ beads/mL, Ø = 4.5 μm, Invitrogen Co., USA) and MyOne magnetic beads with the carboxylic acid functional group (MyOne™ Carboxylic Acid, 6.17 × 10^7^ beads/mL, Ø = 1.0 μm, Invitrogen Co., USA) were used for the cell-bead complexes and for testing the binding selectivity as reported in our previous study^34^,^35^. In brief, 100 μL of the EpiEnrich beads were washed in 1 mL of a 1×phosphate-buffered saline buffer (PBS, Merck Ltd., Darmstadt, Germany) twice, and then were re-suspended into a 1×PBS buffer with a total volume of 1 mL. Next, 10 μL of EpiEnrich beads were loaded with testing cells to perform cell-bead complexes, counted to be 1 × 10^5^ cells/100 μL for the Cell-SELEX process. The binding ratios were also verified by microscope-based measurements. Note that the cell viability test was achieved by using a trypan blue exclusion test^44^ (Trypan blue solution, 0.4%, Sigma-Aldrich Co., USA), and the viability of all tested cell lines were experimentally found to be higher than 85%. Therefore, the screened aptamer targeting agents should be verified to target liable cells.

To determine the binding selectivity of the screened aptamers to the cancer cell lines, the following procedure was conducted. First, 100 μL of MyOne beads were washed twice with 900 μL of ddH_2_O and then the supernatant was removed. Next, 30 μL of amine modified aptamer (100 μM), 20 μL of N-(3-Dimethylaminopropyl)-N’-ethylcarbodiimide hydrochloride (EDAC, 120 mg/mL, Sigma-Aldrich Co., USA), and 950 μL of ddH_2_O were added and incubated for 18 hours at room temperature without light exposure. After repeating the washing step twice with 1 mL of 0.1% of sodium dodecyl sulphate (SDS, Sigma Co., USA) and 0.02% Tween 20 (Sigma-Aldrich Co., USA), 1 mL of ethanol amine (0.1 M, Sigma-Aldrich Co., USA) was applied to block the free uncoupled sites on the surface of the aptamer-magnetic beads at room temperature for one hour without light exposure. After blocking, the aptamer-magnetic beads were washed twice in 1 mL ddH_2_O and re-suspended in 1 mL ddH_2_O and was stored at 4 °C prior to use^34^,^45^,^46^.

### The binding assay for the different rounds of enriched ssDNA pools with flow cytometric analysis

The concentrations of ssDNA in the different rounds of enriched pools were determined by using an ultraviolet-visible wavelengths spectrophotometer (NanoPhotometer® P-Class, Implen GmbH, Germany) and then adjusted to an amount of 50 ng per 2 μL for further PCR amplification. During this PCR process, 5’-Cy5 fluorescence-dye-modified primers (Protech Co., Ltd., Taiwan) were used for PCR amplification. After attaching the Cy5 probe via PCR, the spectrophotometer was used again to determine the concentration of PCR products labelled with the Cy5 probe from the different rounds of pools of enriched ssDNA. The Cy5-probe labelled samples were then adjusted to contain 500 ng per 50 μL for the flow cytometric analysis when binding with CR-CSCs and HCT-8 cells. The fluorescence was further determined by flow cytometry (BD AccuriTM C6, Becton, Dickinson and Company, USA).

For performing the binding assay between the screened aptamers and the cells, 1 × 10^5^ CR-CSCs and HCT-8 cells were incubated in 200 μL of binding buffer with Cy5 probed ssDNA aptamers selected from R1 to R5, respectively, for 30 min at room temperature. The cells were then manually added with 50 μL of washing buffer followed by incubation in the micro-mixer for 1 min. After washing, this step was repeated for another five times. Finally, the Cy5 probed ssDNA bound cells were analyzed using flow cytometry. The relative ssDNA-binding capability of CR-CSC/HCT-8 to the selected aptamers versus the entire ssDNA library could be determined by comparing the results obtained from R1 to R5 as previously described^36^.

### The determination of the dissociation constants from flow cytometric analysis

To determine the binding affinities of the selected aptamers towards CR-CSCs and HCT-8 cells, various concentrations of carboxyfluorescein (FAM) labelled aptamers ranging from 500 nM to 0.1 nM were incubated with CR-CSCs and HCT-8 cells (1 × 10^5^ cells for each), respectively, for 15 min in 50 μL of binding buffer. Cells were then washed six times using 50 μL of washing buffer, and then suspended in 50 μL of binding buffer for analysis via flow cytometry and 1 × 10^4^ cells were counted for analysis. The cell-only samples were also used as the background control. The specific binding intensity was defined by subtracting the mean fluorescence intensity from the background intensity. The resulting fluorescence intensity of the aptamers was further used to calculate the equilibrium dissociation constant (K_d_). K_d_ of the fluorescent aptamer was obtained by using the Prism software (GraphPad Software, Inc. USA) which fitted a plot of the mean fluorescence intensity of the specific binding intensity (Y) versus the aptamer concentration (X), Y = B_max_X/(K_d_ + X) assuming that there was only a biding site^27^,^34^. Note that the fluorescence was determined using the flow cytometer. All of these experiments were repeated three times.

### Fluorescent imaging of immunostaining and CR-CSC/CRC aptamers binding to CR-CSCs and HCT-8 cells

Approximately 1 × 10^5^ CR-CSCs and HCT-8 cells were gently washed with 1 × PBS and then re-suspended in 200 μL of binding buffer before adding 50 μL of 250 nM FAM-labelled aptamer for 30 min of incubation. After incubation, the labelled cells were washed three times with 1 × PBS and fixed in ice-cold 4% paraformaldehyde (Sigma Co., USA) for 5 min, and then air-dried for 5 min. Subsequently, the cells were embedded on microscope slides with ProLong® Gold Antifade Reagent (Invitrogen Co., USA). For immunostaining, CR-CSCs and HCT-8 cells were fixed in ice-cold 4% paraformaldehyde for 30 min, and permeabilized with 0.1% Triton X-100 (Sigma Co., USA) for 10 min. Then the treated cells were washed with 1×PBS for 5 min, three times, and blocked with 1×PBS containing 3% bovine serum albumin (Sigma-Aldrich Co., USA). After washing out the blocking buffer, 1000×diluted primary antibodies were applied for 60-min incubation with treated cells at room temperature. Note that the anti-CD44 antibody (HCAM, H-300, Rabbit, Santa Cruz Biotech., Inc. USA.) was applied for CD44 staining and a CD133 antibody (C24B9, Rabbit, Cell Signaling Tech. Inc. USA) was applied for CD133 staining. After washing out primary antibodies, 100 ×diluted secondary antibody, DyLightTM549-conjugated Goat Anti-Rabbit IgG (Jackson ImmunoResearch Laboratories, Inc. USA), was applied for 30-min incubation. After washing, ProLong® Gold Antifade Reagent was used for embedded cells on slides. The slides were then optically analyzed using a set of components including one collimation lens, one objective lens (Nikon LU Plan 10 × /0.30 A, Nikon, Japan), three fluorescence filters (Nikon G-2A, Nikon, Japan), and a mercury lamp (MODEL C-SHG1, Nikon, Japan). Images of aptamer fluorescence were then captured using a DS-Qi1Mc camera (1.5 megapixels, equipped a Peltier cooling device and a programmable gain amplifier, Nikon, Japan) coupled to an inverted microscope equipped with a digital control module^45^.

### Testing binding selectivity of CR-CSC/CRC aptamers with different types of cancer cells

The binding selectivity test are a measure of the cancer cell capture rate between the selected CR-CSC/HCT-8-specific aptamers with respect to the various cancer cell lines, as mentioned in the previous section. Cancer cells (1 × 10^5^) were incubated with 10 μL of MyOne beads coated with a specific aptamer in the binding buffer for 30 min at a final volume of 200 μL. After incubation, the cancer cells were then washed five times with 200 μL of washing buffer and then suspended in 200 μL of the binding buffer. A side-by-side control test was performed under the same condition but no MyOne beads, counting the bare number of cancer cells after incubation. Next, the captured cells were counted using a hemocytometer under a bright field microscope^34^,^45^.

The capture rate was calculated as follows.


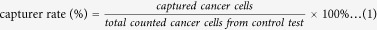


## Additional Information

**How to cite this article**: Hung, L.-Y. *et al.* Screening of aptamers specific to colorectal cancer cells and stem cells by utilizing On-chip Cell-SELEX. *Sci. Rep.* **5**, 10326; doi: 10.1038/srep10326 (2015).

## Supplementary Material

Supplementary Information

## Figures and Tables

**Table 1:** Aptamer sequences of colorectal cancer (CRC) and colorectal cancer stem cells (CR-CSCs) and their corresponding dissociation constants (K_d_).

**Aptamer**	**Aptamer sequence (5’-3’)**^a^	**K**_**d**_ **(nM)**
CSC-16	GGCAGGAAGACAAACAGCGGTCTGACTGGGTAGCAGTAGAAGGCGTCGGTCCCGGGTGGTCTGTGGTGCTGTA	27.4±4.9
CSC-19	TACAGCACCACAGACCACCCGGGACCGACGCCTTCTACTGCTACCCAGTCAGACCGCTGTTTGTCTTCCTGCC	157.2±44.4
CSC-34	GGCAGGAAGACAAACACACAGCCGCACACACAGAACTCCAGGATCTTACCGCCTCATGGTCTGTGGTGCTGTA	48.6±6.8
CSC-35	TACAGCACCACAGACCATGAGGCGGTAAGATCCTGGAGTTCTGTGTGTGCGGCTGTGTGTTTGTCTTCCTGCC	44.8±10.2
HCT-15	GGCAGGAAGACAAACACGCAACAACATACGAAGCCACACAAAAAAAAACACAACCATGGTCTGTGGTGCTGTA	13.2±3.8
HCT-17	TACAGCACCACAGACCATGGTTGTGTTTTTTTTTGTGTGGCTTCGTATGTTGTTGCGTGTTTGTCTTCCTGCC	28.5±8.5
HCT-34	GGCAGGAAGACAAACACCAAAAAAACACCAAAAACAAAACCACTGGTCTGTGGTGCTGTA	12.3±4.5
HCT-35	TACAGCACCACAGACCAGTGGTTTTGTTTTTGGTGTTTTTTTGGTGTTTGTCTTCCTGCC	34.4±6.6

^a^Primer site are underlined

**Table 2:** Comparison of binding selectivity between the commercial antibody and the selected CRC/CR-CSC aptamers against different cancer cell lines.

	**EpiEnrich**^a^	**EpiEnrich**^a^	**CSC−16**	**CSC−19**	**CSC−35**	**CSC−37**	**HCT8–15**	**HCT8–17**	**HCT8–34**	**HCT8–35**
	**One wash**	**Six washes**								
**Cell line**^b^	**Cell capture rate (%)**									
HCT-8	86.0±4.5	34.6±6.6	10.5±2.0	9.0±3.1	10.3±1.5	7.8±1.6	33.3±2.9	47.8±8.7	50.1±1.7	42.5±7.0
CR-CSC	89.3±1.7	68.1±5.1	51.7±5.3	52.9±4.7	45.6±9.1	54.9±4.4	24.7±4.0	18.1±5.4	22.1±4.8	29.1±2.7
A549	96.0±2.2	74.0±4.4	21.2±2.4	28.4±5.7	26.2±1.7	21.2±1.9	23.7±3.1	17.6±5.5	12.7±1.3	14.6±3.3
MCF7	94.1±3.9	66.8±6.8	32.0±6.5	23.2±8.4	29.9±7.8	24.5±0.6	36.1±4.9	34.8±3.5	34.0±5.4	28.1±5.8
BxPC3	70.2±6.8	4.4±1.0	22.2±3.8	20.6±1.1	18.5±3.8	20.1±0.7	23.7±6.9	31.9±5.8	19.7±2.3	17.5±0.8
HepG2	71.9±9.2	6.1±1.5	38.1±4.2	18.1±1.3	21.4±6.9	22.8±7.1	20.1±3.1	23.7±9.3	23.1±5.6	24.2±1.2
HeLa	90.5±5.1	44.4±8.9	17.4±5.0	24.3±3.9	19.7±5.9	18.9±9.0	19.1±8.6	19.3±7.1	16.3±4.5	13.5±7.9
NIH3T3	84.1±1.4	9.4±1.2	23.5±5.2	11.0±3.3	13.1±2.1	9.4±0.4	8.4±2.2	9.1±0.8	13.9±2.5	11.6±1.4

^a^commercial magnetic beads, Epithelial Enriched beads.

^b^A549 (Lung cancer cell line), MCF7 (breast cancer cell line), BxPC3 (pancreas cancer cell line), HepG2 (hepatoma line), HeLa cell (cervical cancer cell line), and NIH3T3 (mouse embryonic fibroblast cell line).

## References

[b1] Siegel R., Ma J. M., Zou Z. H. & Jemal A. Cancer Statistics, 2014. Ca-Cancer J. Clin. 64, 9–29, Doi 10.3322/Caac.21208 (2014). 24399786

[b2] Siegel R., DeSantis C. & Jemal A. Colorectal cancer statistics, 2014. Ca-Cancer J Clin. 64, 104–117, Doi 10.3322/Caac.21220 (2014). 24639052

[b3] Levin B. *et al.* Screening and surveillance for the early detection of colorectal cancer and adenomatous polyps, 2008: A joint guideline from the American Cancer Society, the US Multi-Society Task Force on Colorectal Cancer, and the American College of Radiology. Gastroenterology 134, 1570–1595, DOI 10.1053/j.gastro.2008.02.002 (2008). 18384785

[b4] Quintero E. *et al.* Colonoscopy versus fecal immunochemical testing in colorectal-cancer screening. The New England journal of medicine 366, 697–706, 10.1056/NEJMoa1108895 (2012). 22356323

[b5] Allison J. E., Tekawa I. S., Ransom L. J. & Adrain A. L. A comparison of fecal occult-blood tests for colorectal-cancer screening. The New England journal of medicine 334, 155–159, 10.1056/NEJM199601183340304 (1996). 8531970

[b6] Forones N. M. & Tanaka M. CEA and CA 19-9 as prognostic indexes in colorectal cancer. Hepato-Gastroenterol 46, 905–908 (1999). 10370636

[b7] Al-Shuneigat J. M., Mahgoub S. S. & Huq F. Colorectal carcinoma: nucleosomes, carcinoembryonic antigen and ca 19-9 as apoptotic markers; a comparative study. J. Biomed. Sci. 18, Artn 50, Doi 10.1186/1423-0127-18-50 (2011). PMC3150249 21787404

[b8] Van Cutsem E. *et al.* Cetuximab and Chemotherapy as Initial Treatment for Metastatic Colorectal Cancer. N. Engl. J. Med. 360, 1408–1417, Doi 10.1056/Nejmoa0805019 (2009). 19339720

[b9] Eaves C. J. CANCER STEM CELLS Here, there, everywhere? Nature 456, 581–582, Doi 10.1038/456581a (2008). 19052611

[b10] Bonnet D. & Dick J. E. Human acute myeloid leukemia is organized as a hierarchy that originates from a primitive hematopoietic cell. Nature medicine 3, 730–737 , doi:Doi 10.1038/Nm0797-730 (1997). 9212098

[b11] Ricci-Vitiani L. *et al.* Identification and expansion of human colon-cancer-initiating cells. Nature 445, 111–115, Doi 10.1038/Nature05384 (2007). 17122771

[b12] Nguyen L. V., Vanner R., Dirks P. & Eaves C. J. Cancer stem cells: an evolving concept. Nat. Rev. Cancer 12, 133–143, Doi 10.1038/Nrc3184 (2012). 22237392

[b13] Al-Hajj M., Wicha M. S., Benito-Hernandez A., Morrison S. J. & Clarke M. F. Prospective identification of tumorigenic breast cancer cells. P. Natl. Acad. Sci. USA 100, 3983–3988, DOI 10.1073/pnas.0530291100 (2003). PMC153034 12629218

[b14] Reya T., Morrison S. J., Clarke M. F. & Weissman I. L. Stem cells, cancer, and cancer stem cells. Nature 414, 105–111, Doi 10.1038/35102167 (2001). 11689955

[b15] Barker N. *et al.* Identification of stem cells in small intestine and colon by marker gene Lgr5. Nature 449, 1003–U1001, Doi 10.1038/Nature06196 (2007). 17934449

[b16] Brabletz T., Jung A., Spaderna S., Hlubek F. & Kirchner T. Opinion - Migrating cancer stem cells - an integrated concept of malignant tumour progression. Nat. Rev. Cancer 5, 744-749, Doi 10.1038/Nrc1694 (2005). 16148886

[b17] Dalerba P. *et al.* Phenotypic characterization of human colorectal cancer stem cells. P. Natl. Acad. Sci. USA 104, 10158–10163, DOI 10.1073/pnas.0703478104 (2007). PMC1891215 17548814

[b18] Vaiopoulos A. G., Kostakis I. D., Koutsilieris M. & Papavassiliou A. G. Colorectal Cancer Stem Cells. Stem Cells 30, 363–371, Doi 10.1002/Stem.1031 (2012). 22232074

[b19] Yeung T. M., Gandhi S. C., Wilding J. L., Muschel R. & Bodmer W. F. Cancer stem cells from colorectal cancer-derived cell lines. P. Natl. Acad. Sci. USA 107, 3722–3727, DOI 10.1073/pnas.0915135107 (2010). PMC2840416 20133591

[b20] Haraguchi N. *et al.* CD133(+)CD44(+) population efficiently enriches colon cancer initiating cells. Ann. Surg. Oncol. 15, 2927–2933, DOI 10.1245/s10434-008-0074-0 (2008). 18663533

[b21] Ishii H. *et al.* Characteristics of Colon Cancer-Initiating Cells. Tumor Biol. 29, 68–68 (2008).

[b22] Jordan C. T., Guzman M. L. & Noble M. Cancer Stem Cells. N. Engl. J. Med. 355, 1253–1261, 10.1056/NEJMra061808 (2006). 16990388

[b23] Ailles L. E. & Weissman I. L. Cancer stem cells in solid tumors. Curr. Opin. Biotech. 18, 460–466, DOI 10.1016/j.copbio.2007.10.007 (2007). 18023337

[b24] Magee J. A., Piskounova E. & Morrison S. J. Cancer Stem Cells: Impact, Heterogeneity, and Uncertainty. Cancer Cell 21, 283–296, DOI 10.1016/j.ccr.2012.03.003 (2012). 22439924 PMC4504432

[b25] Tuerk C. & Gold L. Systematic Evolution of Ligands by Exponential Enrichment - Rna Ligands to Bacteriophage-T4 DNA-Polymerase. Science 249, 505–510, DOI 10.1126/science.2200121 (1990). 2200121

[b26] Mendonsa S. D. & Bowser M. T. *In vitro* selection of high-affinity DNA ligands for human IgE using capillary electrophoresis. Anal. Chem. 76, 5387–5392, Doi 10.1021/Ac049857v (2004). 15362896

[b27] Sefah K., Shangguan D., Xiong X. L., O’Donoghue M. B. & Tan W. H. Development of DNA aptamers using Cell-SELEX. Nat. Protoc. 5, 1169–1185, DOI 10.1038/nprot.2010.66 (2010). 20539292

[b28] Sefah K. *et al.* DNA Aptamers as Molecular Probes for Colorectal Cancer Study. Plos One 5, ARTN e14269, DOI 10.1371/journal.pone.0014269 (2010). PMC3000811 21170319

[b29] Li W. M. *et al.* Cell-SELEX-based selection of aptamers that recognize distinct targets on metastatic colorectal cancer cells. Biomaterials 35, 6998–7007, DOI 10.1016/j.biomaterials.2014.04.112 (2014). 24857291

[b30] Mosing R. K., Mendonsa S. D. & Bowser M. T. Capillary electrophoresis-SELEX selection of aptamers with affinity for HIV-1 reverse transcriptase. Anal. Chem. 77, 6107–6112, Doi 10.1021/Ac050836q (2005). 16194066

[b31] Bowser M. T., Mendonsa S. D. & Mosing R. CE-selex: *In vitro* selection of DNA APTAMERS using capillary electrophoresis. Abstr. Pap. Am. Chem. S. 229, U139–U139 (2005).

[b32] Park S. M. *et al.* Selection and elution of aptamers using nanoporous sol-gel arrays with integrated microheaters. Lab. on a chip 9, 1206–1212, Doi 10.1039/B814993c (2009). 19370238

[b33] Qian J. R., Lou X. H., Zhang Y. T., Xiao Y. & Soh H. T. Generation of Highly Specific Aptamers via Micromagnetic Selection. Anal. Chem. 81, 5490–5495, Doi 10.1021/Ac900759k (2009). 19480397 PMC2704263

[b34] Weng C. H. *et al.* An automatic microfluidic system for rapid screening of cancer stem-like cell-specific aptamers. Microfluid Nanofluid 14, 753–765, DOI 10.1007/s10404-012-1095-3 (2013).

[b35] Hung L. Y., Wang C. H., Hsu K. F., Chou C. Y. & Lee G. B. An on-chip Cell-SELEX process for automatic selection of high-affinity aptamers specific to different histologically classified ovarian cancer cells. Lab on a chip 14, 4017–4028, Doi 10.1039/C4lc00587b (2014). 25144781

[b36] Van Simaeys D. *et al.* Study of the Molecular Recognition of Aptamers Selected through Ovarian Cancer Cell-SELEX. Plos One 5, ARTN e13770, DOI 10.1371/journal.pone.0013770 (2010). PMC2967474 21072169

[b37] Srivastava V. K. & Nalbantoglu J. Flow cytometric characterization of the DAOY medulloblastoma cell line for the cancer stem-like phenotype. Cytom. Part A. 73A, 940–948, Doi 10.1002/Cyto.A.20633 (2008). 18773455

[b38] Wang P. *et al.* Identification and Characterization of Cells with Cancer Stem Cell Properties in Human Primary Lung Cancer Cell Lines. Plos One 8, ARTN e57020, DOI 10.1371/journal.pone.0057020 (2013). PMC3587631 23469181

[b39] Wang J. P. *et al.* Selection of phage-displayed peptides on live adherent cells in microfluidic channels. P. Natl. Acad. Sci. USA 108, 6909–6914, DOI 10.1073/pnas.1014753108 (2011). PMC3084056 21486998

[b40] Huang C. J., Lin H. I., Shiesh S. C. & Lee G. B. An integrated microfluidic system for rapid screening of alpha-fetoprotein-specific aptamers. Biosens Bioelectron 35, 50–55, DOI 10.1016/j.bios.2012.02.024 (2012). 22410487

[b41] Weng C. H., Lien K. Y., Yang S. Y. & Lee G. B. A suction-type, pneumatic microfluidic device for liquid transport and mixing. Microfluid Nanofluid 10, 301–310, DOI 10.1007/s10404-010-0669-1 (2011).

[b42] Yang Y. N., Hsiung S. K. & Lee G. B. A pneumatic micropump incorporated with a normally closed valve capable of generating a high pumping rate and a high back pressure. Microfluid Nanofluid 6, 823–833, DOI 10.1007/s10404-008-0356-7 (2009).

[b43] Huang S. B., Wu M. H., Cui Z. F., Cui Z. & Lee G. B. A membrane-based serpentine-shape pneumatic micropump with pumping performance modulated by fluidic resistance. J. Micromech. Microeng. 18, Artn 045008, Doi 10.1088/0960-1317/18/4/045008 (2008).

[b44] Strober W. Trypan blue exclusion test of cell viability. *Current protocols in immunology/edited by* John E. Coligan *…* [*et al.*] **Appendix 3**, Appendix 3B, 10.1002/0471142735.ima03bs21 (2001). 18432654

[b45] Hung L. Y. *et al.* An integrated microfluidic platform for rapid tumor cell isolation, counting and molecular diagnosis. Biomed Microdevices 15, 339–352, DOI 10.1007/s10544-013-9739-y (2013). 23315192

[b46] Keefe A. D., Pai S. & Ellington A. Aptamers as therapeutics. Nat. Rev. Drug. Discov. 9, 537–550, Doi 10.1038/Nrd3141 (2010). 20592747 PMC7097324

