# Supplementary material for: Screening of aptamers specific to colorectal cancer cells and stem cells by utilizing On-chip Cell-SELEX
Source: Sci Rep. 2015 May 22;5:10326. doi: 10.1038/srep10326 (PMC4650677; doi:10.1038/srep10326)
Supplement: Supplementary Information [file srep10326-s1.doc]

Supplemental information

**Screening of aptamers specific to colorectal cancer cells and stem cells by utilizing On-chip Cell-SELEX**

Lien-Yu Hung^1^, Chih-Hung Wang^1^, Yu-Jui Che^1^, Chien-Yu Fu^2^, Hwan-You Chang^2^, Kuan Wang^3^ and Gwo-Bin Lee^1,4,5+^

^1^Department of Power Mechanical Engineering, National Tsing Hua University, Hsinchu, Taiwan 30013

^2^Institute of Biomedical Engineering, National Tsing Hua University, Hsinchu, Taiwan 30013

^3^ Institute of Biological Chemistry, Academia Sinica, Taipei, Taiwan 11529

^4^Institute of NanoEngineering and Microsystems, National Tsing Hua University, Hsinchu, Taiwan 30013

^5^Institute of Molecular Medicine, National Tsing Hua University, Hsinchu, Taiwan 30013

1. The experimental protocol of the on-chip Cell-SELEX

Supplemental Table 1: detailed information about the experimental protocol on the Cell-SELEX microfluidic platform.

| Step | Procedure | Sample volume (μL) | On-chip operation condition |
| --- | --- | --- | --- |
| 1. | Load ssDNA library (1 μM) into the target cell chambers (two open-chamber micromixers/micropumps) | 10 | Turn on heating area at 95℃ for 5 min and then turn it off |
|  | Load the target cell-bound magnetic beads (10^5^/20 μL) into the target cell chambers (two open-chamber micromixers/micropumps) | 20 | Turn on the cooling area to keep the reagents fresh |
|  | Load the control cell-bound magnetic beads (10^5^/20 μL) into the control cell chambers (two open-chamber micromixers/micropumps) | 20 |  |
|  | Load the washing buffer into the washing buffer chamber | 200 |  |
|  | Load the binding buffer into the binding buffer chamber | 200 |  |
|  | Load the PCR reagent into the PCR chambers | 23 |  |
| 2. | Incubate ssDNA library with the target cells for 15 min |  | -40 kPa and 1 Hz for the open-chamber micromixer/micropump |
| 3. | Use a magnet to collect the ssDNA-target cell-magnetic bead complexes onto the surface of the target cell chamber for 1 min |  |  |
| 4. | Remove the incubation supernatant through the waste chamber |  | -80 kPa for the suction pressure |
| 5. | Transport washing buffer into the target cell chambers | 40 | -80 kPa and 0.67 Hz for the transportation unit |
| 6. | Wash the collected ssDNA-target cell-magnetic bead complexes by the open-chamber micromixer/micropump for 1 min |  | Various vacuum pressures (from -40, -50, -60, -70 to -80 kPa) and 1 Hz for the open-chamber micromixer/micropump |
| 7. | Repeat the steps 3-5 for six times |  |  |
| 8. | Use a magnet to collect the ssDNA-target cell-magnetic bead complexes and re-suspend the complexes with binding buffer | 20 | -80 kPa and 0.67 Hz for the transportation unit |
| 9. | Thermal lysis of the target cells and release the bound ssDNA |  | Turn on heating area at 95℃ for 10 min and then turn it off |
| 10. | Transport the bound ssDNA into the control cell chambers for negative selection | 10 | -80 kPa and 15 Hz for the S-shape micropump |
| 11. | Incubate the bound ssDNA with the control cells for 15 min |  | -40 kPa and 1 Hz for the open-chamber micromixer/micropump |
| 12. | Use a magnet to collect the ssDNA-control cell-magnetic bead complexes onto the surface of the control cell chamber for 5 min |  |  |
| 13. | Transport incubation supernatant to the PCR chamber for ssDNA amplification of the selected ssDNA | 2 | -40 kPa and 1 Hz for the open-chamber micromixer/micropump |
| 14. | Final, transport 10 μL of PCR product to the target cell chamber for the next round of CR-CSC Cell-SELEX |  |  |

1. Characterization of the flow pumping rate and the mixing index of the transportation unit and the S-shape micropump

The integrated microfluidic chip was consisted of three layers, including two layers of PDMS and one glass substrate. The first PDMS layer was a thin layer with a thickness of 100 μm, which was a liquid channel layer. Another thick PDMS layer (around 800 μm), which contained air channels, was used to control the micropumps.

The main function of the transportation unit of the microfluidic chip was to transport the binding buffer and the washing buffer to the target cell region. Because the transport process requires three steps, reducing the operating time for each fluid transport step would effectively increase the overall flow rate. The fluidic pumping rate was also found to increase with an increase in the applied gauge pressure (i.e. higher vacuum), as shown in Supplemental Figure 1(a). The maximum pumping rate of the transportation unit was found to be 544.2 μL/min when operated at a frequency of 0.67 Hz at a gauge pressure of -80 kPa.

Similarly, the S-shape micropumps were used to transport the thermally-released ssDNA into the control cell region. However, the S-shape micropumps were operated at a higher driving frequency so that the fluid was more efficiently transported. Furthermore, the 3-cm-long microchannel between the heating and the cooling areas contained 7 μL of fluidic dead volume so that these S-shape micropumps could not transport sufficient reagents under a lower driving frequency. The maximum pumping rate of the S-shape micropump was experimentally found to be 252.7 μL/min when driven at a frequency of 15.0 Hz at a gauge pressure of -80 kPa, as shown in Supplemental Figure 1(b).


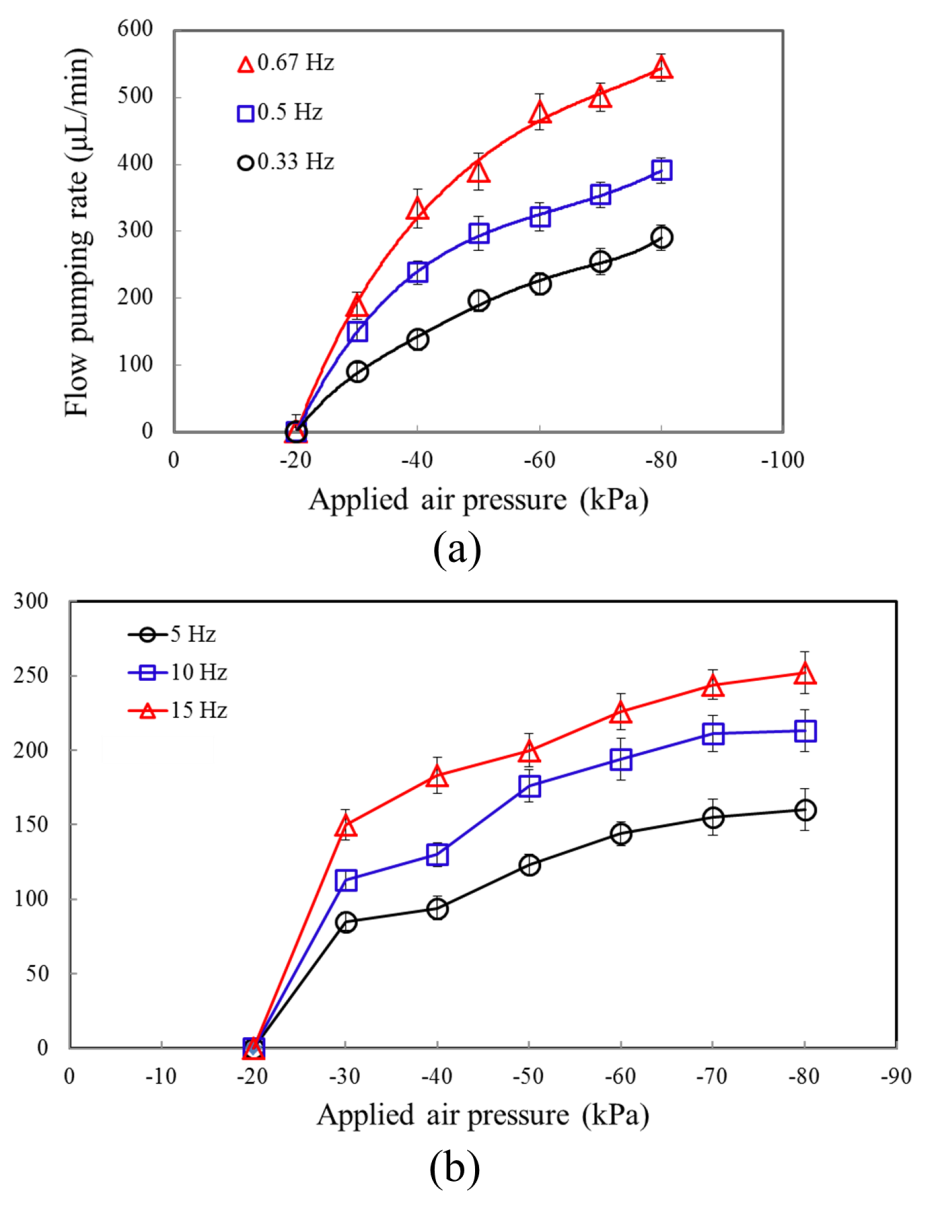


Figure S1. Characterization of the transportation unit and the S-shape micropump for Cell-SELEX of CR-CSC/CRC. (a) The relationship between the pumping rate and the applied gauge pressure for the transportation unit when operated at different driving frequencies. The maximum pumping rate of the transportation unit was experimentally found to be 544.2 μL/min when operated at 0.67 Hz and -80 kPa; (b) The relationship between the pumping rate and the applied gauge pressure for the S-shape micropump when operated at different driving frequencies. The maximum pumping rate was measured to be 252.7 μL/min at 15.0 Hz and -80 kPa;

1. The ssDNA-folding structure of the selected aptamers

Supplemental Figure 2 shows the predicted ssDNA-folding structures of the aptamers at 25℃, using MFOLD software version 3.5 (available at http://mfold.rna.albany.edu/)


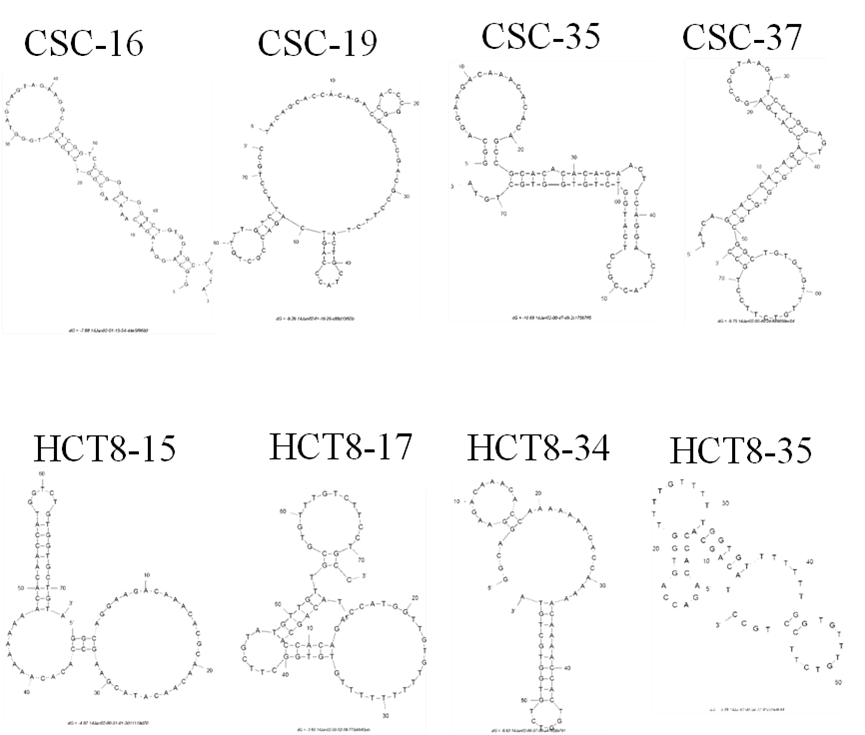


Figure S2. The ssDNA-folding structures of four CR-CSC specific aptamers, including CSC-16, CSC-19, CSC-35 and CSC-37; four HCT-8-specific aptamers, including HCT8-15, HCT8-17, HCT8-34 and HCT8-35, are presented by MFOLD program.

1. The control assay of fluorescence imaging

This control test was performed by incubating FAM labelled CR-CSC aptamers with HCT-8 cells, and FAM labelled HCT-8 aptamers with CR-CSC. Most of them showed weak to no green fluorescence signals, indicating that the selected aptamers could only recognize the target cells.


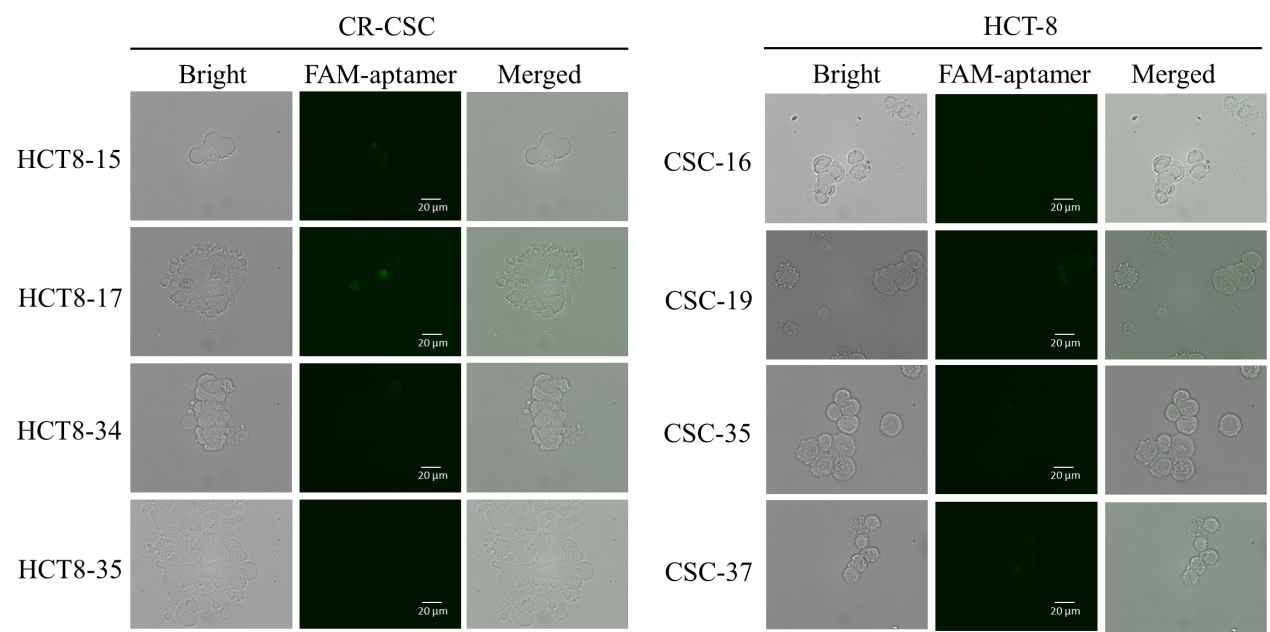


Figure S3. Binding control assay for the selected specific aptamers by using FAM fluorescence labelled aptamers when binding with non-target cells. (a) FAM labelled HCT-8 specific aptamers, HCT8-15, HCT8-17, HCT8-34, and HCT8-35, binding with CR-CSCs; (b) FAM labelled CR-CSC specific aptamers, CSC-16, CSC-19, CSC-35, and CSC-37, binding with HCT-8 cells.
